# Serum cholinesterase is associated with incident diabetic retinopathy: the Shanghai Nicheng cohort study

**DOI:** 10.1186/s12986-023-00743-2

**Published:** 2023-05-03

**Authors:** Rong Yu, Xiaoqi Ye, Xiangning Wang, Qiang Wu, Lili Jia, Keqing Dong, Zhijun Zhu, Yuqian Bao, Xuhong Hou, Weiping Jia

**Affiliations:** 1 Department of Endocrinology and Metabolism, Shanghai Sixth People’s Hospital Affiliated to Shanghai Jiao Tong University School of Medicine, Shanghai Diabetes Institute, Shanghai Key Laboratory of Diabetes Mellitus, Shanghai Clinical Center for Diabetes, Shanghai Key Clinical Center for Metabolic Disease, Shanghai, China; 2 Department of Ophthalmology, Shanghai Sixth People’s Hospital Affiliated to Shanghai Jiao Tong University School of Medicine, Shanghai, China; 3 General Practitioner Teams in Community Health Service Center of Nicheng, Pudong New District, Shanghai, China

**Keywords:** Diabetic retinopathy, Serum cholinesterase, Metabolism, Observational study

## Abstract

**Background:**

Serum cholinesterase (ChE) is positively associated with incident diabetes and dyslipidemia. We aimed to investigate the relationship between ChE and the incidence of diabetic retinopathy (DR).

**Methods:**

Based on a community-based cohort study followed for 4.6 years, 1133 participants aged 55–70 years with diabetes were analyzed. Fundus photographs were taken for each eye at both baseline and follow-up investigations. The presence and severity of DR were categorized into no DR, mild non-proliferative DR (NPDR), and referable DR (moderate NPDR or worse). Binary and multinomial logistic regression models were used to estimate the risk ratio (RR) and 95% confidence interval (CI) between ChE and DR.

**Results:**

Among the 1133 participants, 72 (6.4%) cases of DR occurred. The multivariable binary logistic regression showed that the highest tertile of ChE (≥ 422 U/L) was associated with a 2.01-fold higher risk of incident DR (RR 2.01, 95%CI 1.01-4.00; *P* for trend < 0.05) than the lowest tertile (< 354 U/L). The multivariable binary and multinomial logistic regression showed that the risk of DR increased by 41% (RR 1.41, 95%CI 1.05–1.90), and the risk of incident referable DR was almost 2-fold higher than no DR (RR 1.99, 95%CI 1.24–3.18) with per 1-SD increase of log_e_-transformed ChE. Furthermore, multiplicative interactions were found between ChE and elderly participants (aged 60 and older; *P* for interaction = 0.003) and men (*P* for interaction = 0.044) on the risk of DR.

**Conclusions:**

In this study, ChE was associated with the incidence of DR, especially referable DR. ChE was a potential biomarker for predicting the incident DR.

**Supplementary Information:**

The online version contains supplementary material available at 10.1186/s12986-023-00743-2.

## Introduction

Diabetic retinopathy (DR) is a highly specific microvascular complication of diabetes [1]. DR reached the top 5 leading causes of blindness in adults aged 50 years and above in 2020 according to the report of the vision loss expert group of the Global Burden of Disease Study [2]. While great progress has been made in the treatment of DR in recent years, these treatments are costly, and the related side effects (such as infection, vitreous hemorrhage, etc.) are concerning [3]. With the aging population and the worldwide diabetes epidemic, DR has become and will continue to be a severe public health issue shared globally in the following decades.

Although glycemia, blood pressure, and lipids are traditional modifiable risk factors for DR [3], only 9% of DR progression and 10% of proliferative DR (PDR) incidence could be attributed to these factors reported in the Wisconsin Epidemiology Study of Diabetic Retinopathy (WESDR) [4]. Therefore, it is imperative to actively explore new and reliable biomarkers that can predict DR.

Serum cholinesterase (ChE) is an α glycoprotein synthesized by the liver and has two main forms: acetylcholinesterase (AChE) and butyrylcholinesterase (BChE) [5], and the majority found in human plasma is the latter (BChE: AChE = 412.5: 1) [6]. Clinically, the level of ChE sensitively reflects the synthetic function of the liver [5]. Previous case-control studies reported that serum or plasma ChE levels were significantly higher in participants with diabetes [7], hypertension [7], or diabetic peripheral neuropathy [8]. As for prospective cohort studies, serum BChE increased the risk of incident diabetes among Japanese men [9], and the risk of incident dyslipidemia in participants in a medical check-up center [10].

However, to the best of our knowledge, there have been no prospective population-based cohort studies exploring the relationship between serum ChE and incident DR. Based on a community-based cohort study, we prospectively (1) evaluated the association between baseline serum ChE and the risk of incident DR, including the severity of DR, (2) explored the potential interactions between ChE and metabolic disorders on the incidence of DR, and (3) compared the performances of the known DR predictive models with or without ChE.

## Materials and methods

### Participants and study design

The Shanghai Nicheng Cohort Study was a community-based cohort study. It has been well-documented in previous literature [11]. In brief, the purpose of this cohort was to evaluate the epidemiological characteristics of cardiovascular metabolic diseases in the Chinese population. The baseline survey was launched between April 2013 and August 2014. Individuals aged 55–70 years old at baseline were invited to the follow-up survey in 2018.

The inclusion criteria were met if the participants were 55–70 years old, were diagnosed with diabetes, and had complete fundus photos and ChE data at baseline. The exclusion criteria for the participants included having retinopathy at baseline (including DR, retinitis pigmentosa, hypertensive retinopathy, glaucoma, macular degeneration, and white dot syndrome); taking drugs that affected ChE (including ChE inhibitors, lipid-lowering treatments, and liver-protecting drugs) two weeks before the baseline survey; severe impairment of liver function (alanine aminotransferase [ALT] or aspartate aminotransferase [AST] exceeded 2 times the upper limit of the detection range); having histories of a cardiovascular or malignant tumor at baseline; missing fundus photographs in the follow-up survey. To sum up, we included 2442 participants in this study. 1753 participants finished the follow-up investigation (the follow-up rate was 71.8%). Finally, 1133 participants with diabetes were analyzed in this study (Additional file 1).

### Anthropometric and biochemical indicators

During the baseline and follow-up investigation, the standard questionnaire was used to collect data regarding demographics, lifestyles, disease histories, and other information. The collection of biochemical and anthropometric indicators (including height, weight, and blood pressure) has been described in detail [11]. Current smokers were defined as people who have smoked at least one cigarette a day in the past year. Current drinkers were defined as individuals who had consumed at least 1 g of alcohol per week in the last 1 year. Body mass index (BMI) was the ratio of weight (kg) to the square of height (m).

After at least 10 h of overnight fasting, blood samples were obtained from each participant. The serum ChE was detected using the L-type ChE assay kit (produced by the FUJIFILM Wako Pure Chemical Corporation), with the methylthiophenecarbonylthiocholine as the substrate. The lowest detectable level was 203 U/L and the range of interassay variation was 0.58–1.11%. Fasting plasma glucose (FPG) and 2-h plasma glucose (2-h PG) were tested by the glucose oxidase method. Hemoglobin A1c (HbA1c) was tested by high-performance liquid chromatography. Fasting insulin (FINS) was measured by electrochemiluminescence immunoassay. Triglyceride (TG) and total cholesterol (TC) were determined using the enzymatic colorimetric method. Low-density lipoprotein cholesterol (LDL-C) and high-density lipoprotein cholesterol (HDL-C) were assessed by the direct method. Urine creatinine and urine albumin were tested by the sarcosine oxidase-PAP method and rate nephelometry assay, respectively. ALT, AST, and γ-glutamine transferase (GGT) were determined using the ultraviolet-lactate dehydrogenase method, ultraviolet-malic dehydrogenase method, and γ-glutamyl-p-nitroaniline method, respectively. The calculation formula of homeostasis model assessment-insulin resistance (HOMA-IR) was FINS (mU/L) × FPG (mmol/L) /22.5 [12]. The estimated glomerular filtration rate (eGFR) was computed by the Chronic Kidney Disease Epidemiology Collaboration equation [13].

### Assessment and definition of the outcome

Before the fundus photography, participants were required to rest in a dark room for a moment to allow their pupils to dilate naturally. Non-mydriatic cameras (baseline: Canon CR6 45NM; follow-up: TOPCON TRC-NW400) were used to take photographs of each eye. The photographs were evaluated by two experienced and qualified ophthalmologists who were masked regarding the health status of participants, and inconsistent results were verified by a third ophthalmologist. The DR score was based on their worse eye.

Based on the diagnosis criteria of the American Diabetes Association, participants who had a history of diabetes, FPG ≥ 126 mg/dL, 2-h PG ≥ 200 mg/dL, and/or HbA1c ≥ 6.5% were diagnosed with diabetes [14]. The presence and severity of DR were graded according to the International Clinical Diabetic Retinopathy Disease Severity Scales [15]. Referable DR was defined as moderate non-proliferative DR (NPDR) or worse [16].

### Statistical analysis

The Kolmogorov-Smirnov test showed that the distribution of all continuous variables were skewed. Thus, the continuous variables were shown as median (25th-75th percentile), and the categorical variables were shown as numbers (proportions). The comparisons of the distributions of continuous variables were using the Wilcoxon rank test or Kruskal-Wallis test, and for categorical variables, the chi-square test or Fisher exact test was used as appropriate. To compare the ChE levels among different DR severity groups, least squares mean and standard errors of log_e_-transformed ChE were calculated using the analysis of covariance adjusted for related factors, and the trend was tested using linear regression. The Spearman and partial correlation analyses were used to estimate the correlations between ChE and baseline clinical measurements, respectively. Multivariable binary logistic regression was used to evaluate the risk ratios (RR) and 95% confidence intervals (CI) for the risk of DR with the ChE tertiles. The linear trend between ChE levels and incident DR was tested using the median of ChE in each tertile group as a continuous variable with the binary logistic regression analysis. Meanwhile, we evaluated the effect of per 1-SD increase of log_e_-transformed ChE using both the binary and multinomial logistic regression models. The selection of confounders in the regression models was based on: risk factors of DR; factors related to ChE or DR; biological interests (ALT, reflecting the liver function; HbA1c, directly affecting ChE level). Moreover, We separately conducted the stratified analyses by sex, family history of diabetes, and the cut-off points of age (60 and 65 years old), BMI (24 kg/m^2^), HbA1c (6.5%), TG (150 mg/dL), and LDL-C (100 mg/dL). We also simultaneously included ChE, the aforementioned categorical variables, and their product term in the logistic regression to estimate the multiplicative interaction.

In addition, we evaluated the area under the receiver operating characteristic curve (AUC), integrated discrimination improvement (IDI), relative IDI (rIDI), and net reclassification improvement (NRI) to assess the predictive ability of the models with or without ChE. Both IDI and rIDI could measure the discrimination value of the predictive models and NRI could assess the improvement in the disease reclassification after the addition of ChE [17, 18]. We used two predictive models established previously [19, 20]. Model 1 included sex, diabetes duration, HbA1c, systolic blood pressure (SBP), albuminuria, creatinine clearance, and glucose-lowering treatment [19]. Model 2 included age, diabetes duration, 2-h PG, HbA1c, urine creatinine, albuminuria, and SBP [20].

All the statistical analyses were performed using the SAS software (vision 9.4). A two-tailed *P* < 0.05 was considered statistically significant.

## Results

Among the 1133 individuals with diabetes, the median age was 61.8 years old (25th-75th percentile, 58.9–65.3), and 60.1% were women. Of them, 32.2% had known diagnosed diabetes, and 15.4% had diabetes duration over 5 years (Table 1). Participants in the highest ChE tertile group were younger, and had higher glycemia levels, FINS, HOMA-IR, lipid levels, eGFR, UCAR, ALT, GGT, BMI, and blood pressure levels. Moreover, they had higher proportions of women and glucose-lowering treatment, but a lower proportion of current smokers (Additional file 2: Table S1). Furthermore, the Spearman partial correlation analyses between baseline serum ChE and clinical characteristics showed that after adjustment for age and sex, ChE was moderately correlated with TG, HOMA-IR, and FINS (range of *r* from 0.34 to 0.43). In contrast, ChE was negatively correlated with HDL-C (*r* = -0.16, *P* < 0.001) (Additional file 3: Table S2).

**Table 1:** Baseline characteristics of the study population grouped by incident DR

Characteristics	Total (n = 1133)	No DR (n = 1061)	Incident DR (n = 72)	*P* value
Age, years	61.8 (58.9–65.3)	61.7 (58.8–65.3)	62.6 (59.3–65.6)	0.295
Women, %	681 (60.1)	645 (60.8)	36 (50.0)	0.070
Diabetes duration ≥ 5 years, %	174 (15.4)	155 (14.6)	19 (26.4)	0.008
HbA1c, mmol/mol	46.4 (41.0–53.0)	46.4 (41.0–53.0)	48.6 (41.0–59.0)	0.088
HbA1c, %	6.4 (5.9-7.0)	6.4 (5.9-7.0)	6.6 (5.9–7.6)	-
FPG, mg/dL	129.5 (117.1-147.6)	129.0 (116.4-146.7)	135.0 (124.2-167.7)	0.002
FINS, uU/mL	8.3 (5.7–12.2)	8.2 (5.7–12.0)	8.4 (5.7–13.3)	0.352
HOMA-IR	2.6 (1.8–4.1)	2.6 (1.8–4.1)	3.0 (2.0–5.0)	0.053
TC, mg/dL	203.9 (180.3-230.9)	203.9 (179.9-231.7)	201.2 (182.6-223.7)	0.689
LDL-C, mg/dL	122.8 (103.5-144.4)	123.2 (104.2-144.8)	114.9 (95.0-140.2)	0.094
HDL-C, mg/dL	48.6 (40.9–57.5)	48.6 (40.9–57.5)	48.8 (37.8–60.2)	0.976
TG, mg/dL	136.3 (96.5-212.4)	136.3 (95.6-208.8)	140.7 (106.2-217.3)	0.124
eGFR, mL/min/1.73 m^2^	96.3 (91.1-100.7)	96.2 (90.8-100.7)	97.1 (93.0-100.3)	0.255
UACR, mg/g	7.9 (5.1–14.3)	7.7 (5.0-13.9)	9.4 (6.0-20.3)	0.026
ALT, U/L	19.0 (14.0–26.0)	19.0 (14.0–26.0)	20.0 (15.0–28.0)	0.297
AST, U/L	22.0 (19.0–27.0)	22.0 (19.0–27.0)	22.0 (20.0–27.0)	0.783
GGT, U/L	29.0 (20.0–45.0)	29.0 (20.0–45.0)	30.5 (21.0–49.0)	0.274
BMI, kg/m^2^	25.8 (23.6–27.8)	25.8 (23.5–27.8)	26.1 (24.6–27.9)	0.240
SBP, mmHg	135.0 (127.0-147.0)	135.0 (127.0-147.5)	132.0 (126.0-140.0)	0.068
DBP, mmHg	83.0 (80.0–89.0)	83.0 (80.0–90.0)	83.0 (80.0-86.8)	0.828
Current smoker, %	201 (17.7)	191 (18.0)	10 (13.9)	0.377
Current drinker, %	167 (14.7)	147 (13.9)	20 (27.8)	0.001
Physical activity ≥ 30 min/day, %	40 (3.5)	38 (3.6)	2 (2.8)	1.000
Family history of diabetes, %	246 (21.8)	221 (20.9)	25 (34.7)	0.006
Glucose-lowering treatment, %	270 (23.8)	244 (23.0)	26 (36.1)	0.012

Data were n (%) for categorical measures or median (25th-75th percentile) for continuous measures

Abbreviations: DR, diabetic retinopathy; ChE, cholinesterase; HbA1c, hemoglobin A1c; FPG, fasting plasma glucose; 2-h PG, 2-h plasma glucose; FINS, fasting insulin; HOMA-IR, homeostasis model assessment-insulin resistance; TC, total cholesterol; LDL-C, low-density lipoprotein cholesterol; HDL-C, high-density lipoprotein cholesterol; TG, triglyceride; eGFR, estimated glomerular filtration rate; UACR, urinary albumin creatinine ratio; ALT, alanine aminotransferase; AST, aspartate aminotransferase; GGT, γ-glutamine transferase; BMI, body mass index; SBP, systolic blood pressure; DBP, diastolic blood pressure

**Fig. 1:**
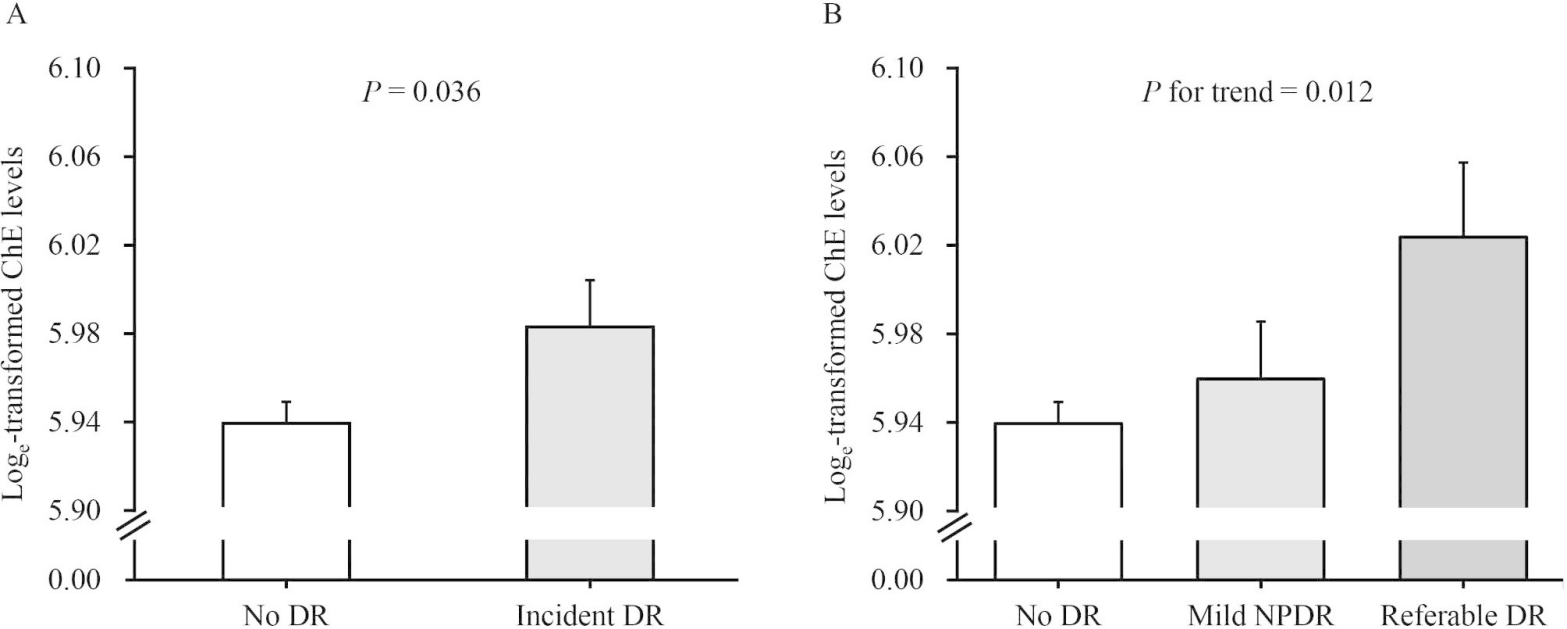
The least-square means of ChE by the presence and severity of DR groups. The least-square means of log_e_-transformed ChE with standard errors were presented as dot and error bars after adjustment for age, sex, BMI, SBP, smoking and drinking status, family history of diabetes, duration of diabetes, glucose-lowering treatment, HbA1c, ALT, TG, LDL-C, and eGFR. Abbreviations: DR, diabetic retinopathy; ChE, cholinesterase; RR, risk ratio; CI, confidence interval; BMI, body mass index; SBP, systolic blood pressure; HbA1c, hemoglobin A1c; ALT, alanine aminotransferase; TG, triglyceride; LDL-C, low-density lipoprotein cholesterol; eGFR, estimated glomerular filtration rate

**Fig. 2:**
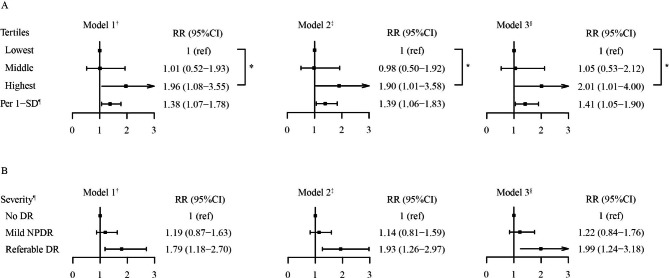
ChE and risks of incidence and severity of DR. (A) ChE and incidence of DR using the binary logistic regression. (B) ChE and severity of DR using the multinomial logistic regression. ^*^P for trend was tested using the median of each group as a continuous variable in the logistic regression. All *P* for trend was < 0.05. ^†^ Model 1 was adjusted for age and sex. ^‡^ Model 2 was adjusted for age, sex, BMI, SBP, smoking and drinking status, family history of diabetes, diabetes duration, and glucose-lowering treatment. ^§^ Model 3 was adjusted for age, sex, BMI, SBP, smoking and drinking status, family history of diabetes, duration of diabetes, glucose-lowering treatment, HbA1c, ALT, TG, LDL-C, and eGFR. ^¶^ The effect was scaled by the 1-SD increase of log_e_-transformed ChE. Abbreviations: DR, diabetic retinopathy; ChE, cholinesterase; RR, risk ratio; CI, confidence interval; BMI, body mass index; SBP, systolic blood pressure; HbA1c, hemoglobin A1c; ALT, alanine aminotransferase; TG, triglyceride; LDL-C, low-density lipoprotein cholesterol; eGFR, estimated glomerular filtration rate

After a mean 4.6-year follow-up, 72 (6.4%) were diagnosed with incident DR, of which 45 were mild NPDR (4.0%) and 27 were referable DR (2.4%). The baseline ChE level was significantly higher in the participants with incident DR (*P* = 0.036), particularly in individuals with referable DR (*P* for trend = 0.012) (Fig. 1). In addition, the multivariable logistic regression analyses showed that baseline serum ChE was positively associated with incident DR, particularly referable DR (Fig. 2). In the binary logistic regression analyses, compared with the lowest ChE tertile group, the highest ChE tertile group had a 2.01-fold higher risk of incident DR (RR 2.01, 95%CI 1.01-4.00). A linear association was found between ChE and DR risk (*P* for trend < 0.05). In addition, each 1-SD increase of log_e_-transformed ChE was associated with a 41% higher risk of incident DR (RR 1.41, 95%CI 1.05–1.90) (Fig. 2A). Furthermore, in the multinomial logistic regression analyses, the higher level of ChE was associated with a higher risk of referable DR (RR 1.99, 95%CI 1.24–3.18) compared with the no DR group, but not significant in the mild NPDR group (RR 1.22, 95% CI 0.84–1.76) (Fig. 2B).

**Fig. 3:**
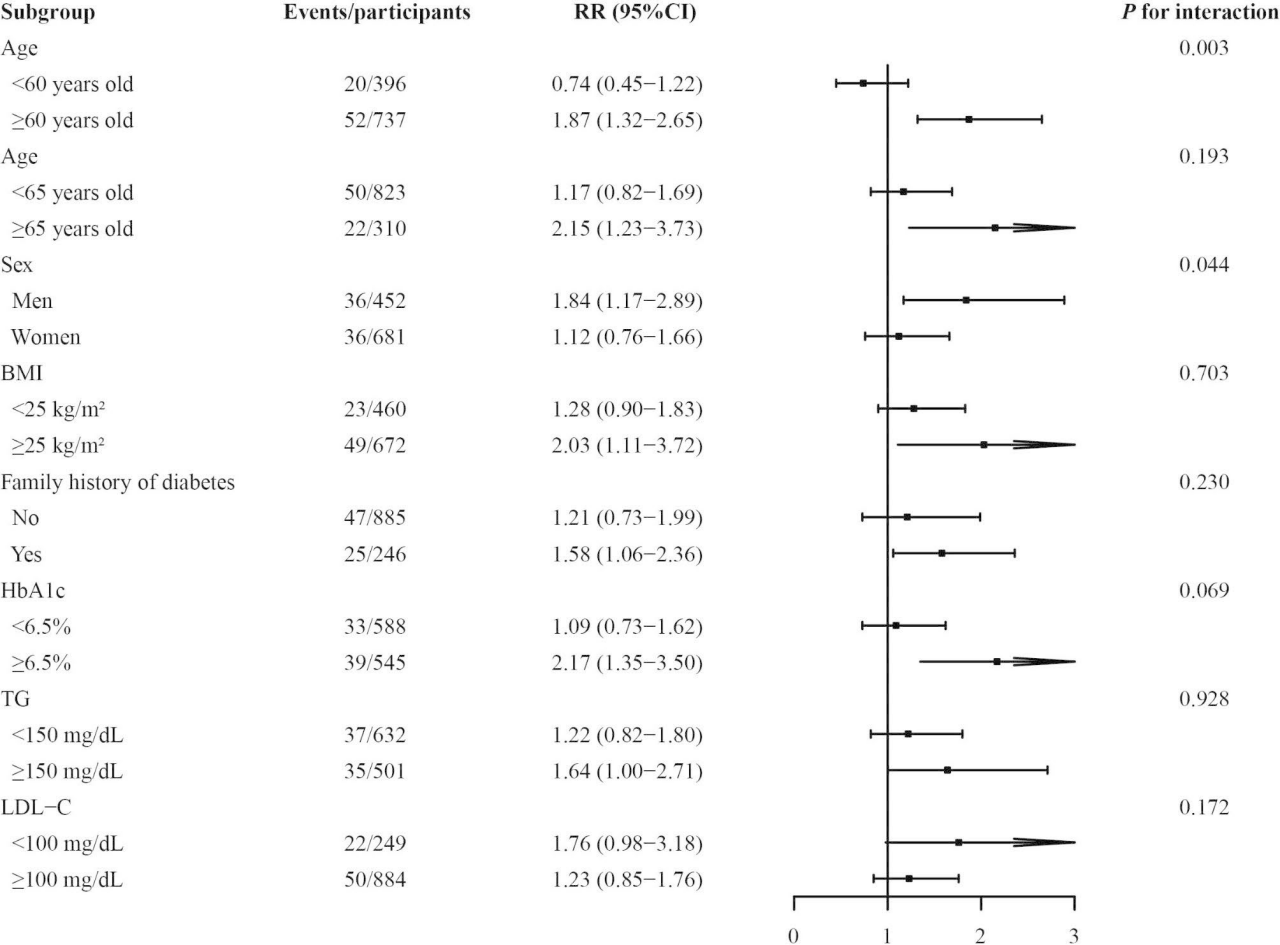
Stratified analyses of the risk of incident DR according to per 1-SD increase of loge-transformed ChE. Models were adjusted for age, sex, BMI, SBP, smoking and drinking status, family history of diabetes, duration of diabetes, glucose-lowering treatment, HbA1c, ALT, TG, LDL-C, and eGFR. Abbreviations: DR, diabetic retinopathy; ChE, cholinesterase; RR, risk ratio; CI, confidence interval; BMI, body mass index; SBP, systolic blood pressure; HbA1c, hemoglobin A1c; ALT, alanine aminotransferase; TG, triglyceride; LDL-C, low-density lipoprotein cholesterol; eGFR, estimated glomerular filtration rate

Stratified analyses of two strata showed that the associations between ChE and incident DR were significantly positive in the strata of men, with a family history of diabetes, with age ≥ 65 years old, BMI ≥ 25 kg/m^2^, HbA1c ≥ 6.5%, or TG ≥ 150 mg/dL, but not significant in their corresponding other strata. However, significant multiplicative interactions of ChE with sex and ageing (cutoff point: 60) on incident DR were found (*Ps* for interaction: 0.044 and 0.003) (Fig. 3).

Finally, there were no significant differences in the AUCs of the models before or after adding ChE to the established models (model 1: 0.65 [95% CI 0.59–0.71], model 1 + ChE: 0.67 [95% CI 0.60–0.73], *P* = 0.320; model 2: 0.65 [95% CI 0.60–0.71], model 2 + ChE: 0.66 [95% CI 0.60–0.72], *P* = 0.686). However, with additional ChE in the two models, IDI, rIDI, and NRI were improved: both IDI in the two models increased by 0.01; model 1, rIDI (0.36, *P* = 0.024) and NRI (0.31, *P* = 0.010); model 2, rIDI (0.54, *P* = 0.019) and NRI (0.27, *P* = 0.025) (Table 2).

**Table 2:** Predictive performance of models for incident DR with or without ChE

	AUC			IDI				NRI	
	AUC (95% CI)	*P* value		IDI	rIDI	*P* value		NRI	*P* value
Model 1^†^	0.65 (0.59–0.71)			Ref.	Ref.			Ref.	
+ ChE	0.67 (0.60–0.73)	0.320		0.01	0.36	0.024		0.31	0.010
Model 2^‡^	0.65 (0.60–0.71)			Ref.	Ref.			Ref.	
+ ChE	0.66 (0.60–0.72)	0.686		0.01	0.54	0.019		0.27	0.025

ChE was each 1-SD log_e_-transformed. Model 1 and model 2 were predictive models established in previous studies

^†^ Independent variables included sex, diabetes duration, HbA1c, SBP, albuminuria, creatinine clearance, and glucose-lowering treatment

^‡^ Independent variables included age, diabetes duration, 2 h-PG, HbA1c, UCR, albuminuria, and SBP

Abbreviations: Ref, reference; ChE, cholinesterase; DR, diabetic retinopathy; IDI, integrated discrimination improvement; rIDI, relative integrated discrimination improvement; NRI, net reclassification improvement; HbA1c, hemoglobin A1c; SBP, systolic blood pressure; 2 h-PG, 2 h- postprandial blood glucose; UCR, urinary creatinine

## Discussion

To our knowledge, this was the first prospective observational cohort study to investigate the serum ChE levels and incident DR. After a mean 4.6-year follow-up, in the middle-aged and older Chinese people with diabetes, higher ChE levels were associated with higher risks of incident DR and referable DR: a 2.01-fold higher risk of DR in the highest tertile versus the lowest tertile, 1.41-fold higher DR risk and an almost 2-fold higher risk of referable DR per 1-SD increase of log_e_-transformed ChE. Moreover, both the male sex and the elderly aged 60 and over had significantly stronger associations between ChE and incident DR than their counterparts. Furthermore, ChE improved the IDI, rIDI, and NRI of the established predictive models, thus, the predictive models with the addition of ChE had better discrimination and significant improvement in diabetic retinopathy reclassification.

The WESDR study indicated that only 9% of the development of DR and 10% of the incidence of PDR were attributed to HbA1c, blood pressure, and TC [4]. Our study demonstrated that when ChE was taken into account with traditional risk factors in the two established models, the predictive abilities improved, with both the IDI improving by 0.01, rIDI improving by 0.36 and 0.54, and the NRI improving by 0.31 and 0.27, respectively [19, 20]. The incremental predictive ability possessed by ChE may be explained by both metabolic and neurological aspects. Consistent with several previous population-based studies [21, 22], we observed that higher ChE was accompanied by worse metabolic profiles: TG, HOMA-IR, and FINS were moderately correlated with ChE. Meanwhile, it has been proposed that DR is not only regarded as a microvascular but also a neurodegenerative disease [23]. Interestingly, recent research revealed that ChE was involved in the process of neurological diseases [24]. Hence, ChE could be regarded as an integrative factor that reflects both the metabolic status and the function of the nervous system.

In particular, another important finding was that elevated ChE was associated with referable DR. As stated by some organizations, including the Chinese Diabetes Society, the presence of referable DR required interventions from professional ophthalmologists [16, 25]. However, with the global epidemic of diabetes, ophthalmology services are overwhelmed. Although the technologies of artificial intelligence have developed in detecting DR in recent years, it still takes time to be widely promoted [26, 27]. Also, the current treatments are mainly for those with advanced stages of DR, and they may not be effective for everyone [3]. Therefore, our study suggested that the testing of ChE, a clinically easily detectable indicator, could facilitate the early identification of people at high risk of developing referable DR. The sooner an individual with high risk is identified, the sooner interventions can be made, and the less likely it is the person will face the dilemma of blindness. Further observational studies with larger samples are still needed to explore the relationship between ChE and more severe DR stages, like vision-threatening DR (severe NPDR or worse) or even PDR.

The association between ChE and DR did not attenuate even after adjustment for TG, LDL-C, and HbA1c in the regression model, which indicated that there were other possible mechanisms independent of glucose and lipid profiles. The substrates of ChE may be used to explain related mechanisms. ChE could hydrolyze acetylcholine (ACh), and BChE could hydrolyze ghrelin specifically [28, 29]. By hydrolyzing ACh, ChE may influence retinal neuron function [30], blood flow conditions [31, 32], and retinal inflammation [33–35]. Moreover, by hydrolyzing ghrelin, BChE could lessen its retinal protective effects, like inhibiting hyperglycemia-induced cell death, reducing the production of reactive oxygen species, and protecting retinal function and structure [36]. On the other hand, in animal experiments, the application of ChE inhibitors could prevent the decrease in retinal thickness and significantly improve retinal blood flow [30, 32]. Nonetheless, the potential mechanisms between ChE and DR have not been fully elaborated. Further interventional trials should be actively conducted to target ChE on preventing or delaying DR, which should be expected to relieve the burdens from both the personal perspective and the social perspective.

The causal role of hyperglycemia in DR has been demonstrated by numerous large-scale cohort studies [1]. Besides, some population-based observational studies reported that ChE was positively correlated with glucose profiles [21, 22]. Also, an in vitro study using the serum from healthy controls showed that the ChE activity elevated with increasing glucose gradients [7]. These all suggested that higher ChE often accompanied higher blood glucose levels. A randomized control trial revealed that the ChE inhibitor galantamine could lower the level of TNF, a molecule that contributed to insulin resistance, and also significantly lower plasma insulin and HOMA-IR in patients with metabolic syndrome [37]. This trial suggested that ChE might be involved in insulin resistance. However, the multiplicative interaction was not statistically significant. This was probably due to the small cases in each subgroup. Further studies are needed to evaluate how metabolic indicators including HbA1c interact with ChE in developing DR.

Although age was reported to not be associated with cholinesterase levels [38], aging itself could lead to changes in the retina, such as reduced blood flow and retinal thinning [39]. In this study, the interaction between ChE and age was only observed in participants aged 60 and older, but not in those aged 65 and older. The small cases may contribute to this result. More studies are needed to elaborate on our results.

Besides, this study revealed that men had statistically significant multiplicative interaction with the association between ChE and incident DR. Intriguingly, consistent with previous studies, among participants with age over 55, ChE was higher in women than men [40]. However, our results showed that men had higher FPG and diastolic blood pressure, lower FINS, worse renal function, and had less healthy lifestyles with respect to smoking, drinking, and physical activity (Additional file 4: Table S3). These adverse factors were reported to be correlated with DR [3]. Meanwhile, men seemed to be more susceptible to DR than women. In individuals with diabetes but without DR, women had significantly lower concentrations of inflammation-associated proteins in aqueous humor than men [41]. Moreover, a case-control study based on 70 adults with type 2 diabetes found that characteristics of neuroretinal function were more abnormal in men than women, like the delayed implicit time of the retinal locations [42]. All in all, the variation in the pathophysiological mechanism of DR between women and men remains an essential area for future research.

This is the first community-based cohort study to explore the association between ChE and incident DR. Fundus photographs were reviewed by professional and experienced ophthalmologists, so the diagnosis of DR was objective and reliable. Our limitations in this study are as follows: first, our study was conducted on Chinese middle-aged and elderly people, so the extrapolation of the findings is limited. Second, we did not identify sufficient cases of DR because of the relatively short follow-up period. Consequently, our results should be interpreted with caution.

In this study, we found that higher levels of serum ChE increased the risk of DR. ChE may be a promising biomarker for DR prevention, particularly referable DR. We could better pinpoint the population at high risk for DR when ChE was taken into account. Further studies are needed to further validate the causality between serum ChE of DR, and confirm whether ChE could be a potential therapeutic target for DR.

## Electronic supplementary material

Below is the link to the electronic supplementary material.

Supplementary Material

## Data Availability

The datasets analysed during the current study are not publicly available due the privacy constraints, but are available from the corresponding author on reasonable request.

## List of abbreviations

ChE: cholinesterase
DR: diabetic retinopathy
PDR: proliferative diabetic retinopathy
NPDR: non-proliferative diabetic retinopathy
AChE: acetylcholinesterase
BChE: butyrylcholinesterase
ALT: alanine aminotransferase
AST: aspartate aminotransferase
BMI: body mass index
FPG: fasting plasma glucose
2-h PG: 2-h plasma glucose
HbA1c: hemoglobin A1c
FINS: fasting insulin
TG: triglyceride
TC: total cholesterol
LDL-C: low-density lipoprotein cholesterol
HDL-C: high-density lipoprotein cholesterol
GGT: γ-glutamine transferase
HOMA-IR: homeostasis model assessment-insulin resistance
eGFR: estimated glomerular filtration rate
UACR: urinary albumin creatinine ratio
SBP: systolic blood pressure
RR: risk ratios
CI: confidence intervals
AUC: area under the receiver operating characteristic curve
rIDI: relative integrated discrimination improvement
NRI: net reclassification improvement
SBP: systolic blood pressure.
