# Supplementary material for: Serum cholinesterase is associated with incident diabetic retinopathy: the Shanghai Nicheng cohort study
Source: Nutr Metab (Lond). 2023 May 3;20:26. doi: 10.1186/s12986-023-00743-2 (PMC10155425; doi:10.1186/s12986-023-00743-2)
Supplement: Supplementary file 1 — Supplementary Material [file 12986_2023_743_MOESM1_ESM.docx]

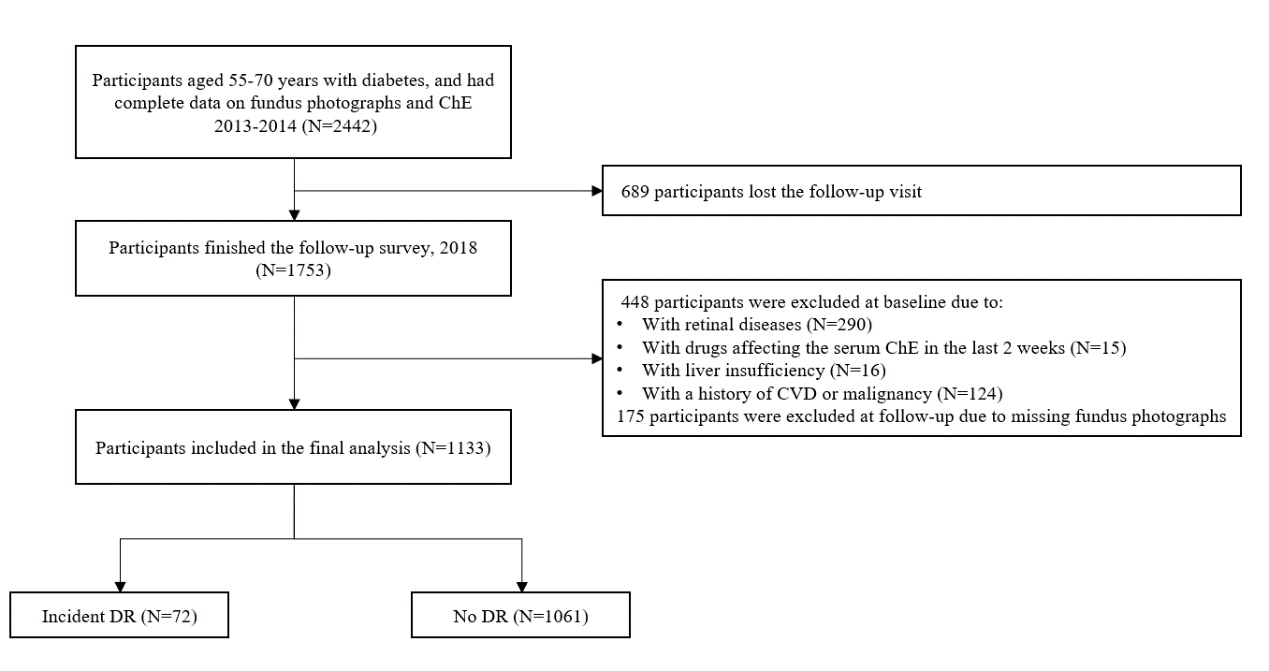


**Additional file 1: Figure S1 | Flowchart of the inclusion and exclusion of the study population.**

Abbreviations: ChE, cholinesterase. CVD, cardiovascular diseases. DR, diabetic retinopathy.

**Additional file 2:Table S1 | Baseline characteristics of the study population grouped by ChE tertiles.**

| Characteristics | Serum ChE levels |  |  |  |
| --- | --- | --- | --- | --- |
|  | Tertile 1 (n=369) | Tertile 2 (n=386) | Tertile 3 (n=376) | *P* value |
| Range of ChE, U/L | <354 | 354-< 422 | ≥ 422 |  |
| Age, years | 62.8 (59.7-66.4) | 61.9 (58.7-65.2) | 61.1 (58.5-64.5) | <0.001 |
| Women, % | 183 (49.6) | 243 (63.0) | 255 (67.5) | <0.001 |
| Diabetes duration ≥5 years, % | 49 (13.3) | 58 (15.1) | 67 (17.7) | 0.238 |
| HbA1c, % | 6.1 (5.7-6.8) | 6.4 (5.9-7.0) | 6.5 (6.0-7.5) | <0.001 |
| FPG, mg/dL | 126.8 (114.2-140.7) | 129.7 (117.1-144.3) | 132.1 (120.5-159.5) | <0.001 |
| 2-h PG, mg/dL | 228.6 (200.7-276.9) | 246.3 (208.3-299.1) | 250.6 (211.0-318.2) | <0.001 |
| FINS, uU/mL | 6.3 (4.3-9.2) | 8.4 (5.9-12.3) | 10.4 (7.1-14.0) | <0.001 |
| HOMA-IR | 2.1 (1.3-3.0) | 2.7 (1.9-4.1) | 3.4 (2.3-5.2) | <0.001 |
| TC, mg/dL | 188.0 (164.9-213.5) | 206.0 (185.7-229.7) | 215.8 (191.9-244.8) | <0.001 |
| LDL-C, mg/dL | 112.4 (94.2-129.7) | 127.8 (108.9-146.3) | 129.3 (110.4-155.2) | <0.001 |
| HDL-C, mg/dL | 50.2 (42.9-61.0) | 47.9 (41.3-56.0) | 47.1 (39.4-56.4) | 0.001 |
| TG, mg/dL | 106.2 (74.3-147.8) | 138.5 (105.3-202.7) | 183.2 (124.8-277.9) | <0.001 |
| eGFR, mL/min/1.73 m^2^ | 94.9 (89.8-99.4) | 96.3 (91.7-100.4) | 97.6 (92.4-102.1) | <0.001 |
| UACR, mg/g | 6.7 (4.6-11.2) | 7.4 (5.2-12.9) | 9.2 (5.8-18.4) | <0.001 |
| ALT, U/L | 17.0 (13.0-22.0) | 19.0 (14.0-27.0) | 21.0 (15.0-30.0) | <0.001 |
| AST, U/L | 22.0 (19.0-26.0) | 22.0 (19.0-27.0) | 23.0 (19.0-27.0) | 0.204 |
| GGT, U/L | 25.0 (17.0-39.0) | 29.0 (21.0-46.0) | 32.0 (22.0-49.0) | <0.001 |
| BMI, kg/m^2^ | 24.6 (22.5-26.6) | 26.1 (24.0-28.1) | 26.7 (24.5-28.5) | <0.001 |
| SBP, mmHg | 133.0 (125.0-145.0) | 135.0 (127.0-147.0) | 138.0 (128.0-148.0) | 0.054 |
| DBP, mmHg | 82.0 (80.0-89.0) | 83.0 (80.0-89.0) | 84.0 (80.0-90.0) | 0.001 |
| Current smoker | 84 (22.8) | 61 (15.8) | 56 (14.8) | 0.008 |
| Current drinker | 66 (17.9) | 54 (14.0) | 47 (12.4) | 0.096 |
| Physical activity ≥30 minutes/day, % | 9 (2.4) | 13 (3.4) | 18 (4.8) | 0.222 |
| Family history of diabetes, % | 72 (19.6) | 86 (22.3) | 88 (23.3) | 0.437 |
| Glucose-lowering treatment, % | 67 (18.2) | 96 (24.9) | 107 (28.3) | 0.004 |

Data were n (%) for categorical measures or median (25th-75th percentile) for continuous measures.

Abbreviations: DR, diabetic retinopathy; ChE, cholinesterase; HbA1c, hemoglobin A1c; FPG, fasting plasma glucose; FINS, fasting insulin; HOMA-IR, homeostasis model assessment-insulin resistance; TC, total cholesterol; LDL-C, low-density lipoprotein cholesterol; HDL-C, high-density lipoprotein cholesterol; TG, triglyceride; eGFR, estimated glomerular filtration rate; UACR, urinary albumin creatinine ratio; ALT, alanine aminotransferase; AST, aspartate aminotransferase; GGT, γ-glutamine transferase; BMI, body mass index; SBP, systolic blood pressure; DBP, diastolic blood pressure.

**Additional file 3:Table S2 | Correlations of serum ChE levels with biochemical and anthropometric characteristics at baseline.**

| Characteristics | Serum ChE levels | | | |
| --- | --- | --- | --- | --- |
|  | *r* | *P* | Adjusted *r* ^†^ | *P* |
| HbA1c (mmol/mol) | 0.22 | <0.001 | 0.21 | <0.001 |
| FPG (mmol/L) | 0.17 | <0.001 | 0.18 | <0.001 |
| FINS (uU/ml) | 0.35 | <0.001 | 0.34 | <0.001 |
| HOMA-IR | 0.38 | <0.001 | 0.36 | <0.001 |
| LDL-C (mmol/L) | 0.26 | <0.001 | 0.24 | <0.001 |
| HDL-C (mmol/L) | -0.12 | <0.001 | -0.16 | <0.001 |
| TC (mmol/L) | 0.32 | <0.001 | 0.30 | <0.001 |
| TG (mmol/L) | 0.43 | <0.001 | 0.43 | <0.001 |
| eGFR (mL/min/1.73 m^2^) | 0.14 | <0.001 | 0.06 | 0.044 |
| UACR (mg/g) | 0.19 | <0.001 | 0.17 | <0.001 |
| GGT (U/L) | 0.22 | <0.001 | 0.27 | <0.001 |
| ALT (U/L) | 0.20 | <0.001 | 0.21 | <0.001 |
| AST (U/L) | 0.07 | 0.014 | 0.08 | 0.005 |
| BMI (kg/m^2^) | 0.27 | <0.001 | 0.28 | <0.001 |
| SBP (mmHg) | 0.08 | 0.008 | 0.09 | 0.003 |
| DBP (mmHg) | 0.11 | <0.001 | 0.13 | <0.001 |
| HR (beats/minute) | 0.04 | 0.171 | 0.03 | 0.303 |

Data were Spearman and Spearman partial coefficient and their corresponding *P* value.

^†^ *r* was adjusted for age and sex.

Abbreviations: ChE, cholinesterase; HbA1c, hemoglobin A1c; FPG, fasting plasma glucose; FINS, fasting insulin; HOMA-IR, homeostasis model assessment-insulin resistance; LDL-C, low-density lipoprotein cholesterol; HDL-C, high-density lipoprotein cholesterol; TC, total cholesterol; TG, triglyceride; eGFR, estimated glomerular filtration rate; UACR, urinary albumin creatinine ratio; GGT, γ-glutamine transferase; ALT, alanine aminotransferase; AST, aspartate aminotransferase; BMI, body mass index; SBP, systolic blood pressure; DBP, diastolic blood pressure; HR, heart rate.

**Additional file 4:Table S3 | Baseline characteristics of the study population grouped by gender.**

| Characteristics | Men (n=452) | Women (n=681) | *P* value |
| --- | --- | --- | --- |
| Age, years | 61.5 (58.6-65.04) | 61.9 (59.2-65.67) | 0.101 |
| Diabetes duration ≥5 years, % | 75 (16.6) | 99 (14.6) | 0.358 |
| ChE, U/L | 373.0 (328.5-427.00) | 397.0 (351.0-447.00) | <0.001 |
| HbA1c, % | 6.3 (5.8-7.20) | 6.4 (5.9-7.00) | 0.296 |
| FPG, mg/dL | 130.5 (119.9-149.64) | 127.9 (116.0-146.13) | 0.022 |
| FINS, uU/mL | 7.3 (4.8-10.76) | 8.9 (6.1-13.09) | <0.001 |
| HOMA-IR | 2.4 (1.6-3.70) | 2.8 (2.0-4.38) | <0.001 |
| TC, mg/dL | 193.2 (167.4-223.36) | 210.0 (187.6-233.98) | <0.001 |
| LDL-C, mg/dL | 116.0 (94.6-139.19) | 126.6 (109.3-149.42) | <0.001 |
| HDL-C, mg/dL | 44.6 (38.6-53.67) | 51.0 (43.2-59.07) | <0.001 |
| TG, mg/dL | 131.0 (88.5-217.26) | 140.7 (101.8-207.08) | 0.087 |
| eGFR, mL/min/1.73 m^2^ | 95.3 (89.7-100.21) | 97.0 (92.2-100.91) | 0.002 |
| UACR, mg/g | 6.2 (4.2-10.52) | 8.7 (5.8-15.86) | <0.001 |
| ALT, U/L | 21.0 (15.0-28.00) | 18.0 (14.0-24.00) | <0.001 |
| AST, U/L | 23.0 (19.0-28.00) | 22.0 (19.0-27.00) | 0.120 |
| GGT, U/L | 37.5 (25.0-54.50) | 25.0 (18.0-38.00) | <0.001 |
| BMI, kg/m^2^ | 26.0 (23.8-27.81) | 25.7 (23.4-27.86) | 0.321 |
| SBP, mmHg | 135.0 (127.0-146.00) | 135.0 (127.0-148.00) | 0.974 |
| DBP, mmHg | 84.0 (80.0-90.00) | 82.0 (80.0-88.50) | <0.001 |
| Current smoker | 201 (44.5) | 0 (0.00) | <0.001 |
| Current drinker | 161 (35.6) | 6 (0.88) | <0.001 |
| Physical activity ≥30 minutes/day, % | 15 (3.3) | 25 (3.7) | 0.752 |
| Family history of diabetes, % | 96 (21.2) | 150 (22.1) | 0.734 |
| Glucose-lowering treatment, % | 107 (23.7) | 163 (23.9) | 0.919 |

Data were n (%) for categorical measures or median (25th-75th percentile) for continuous measures.

Abbreviations: DR, diabetic retinopathy; ChE, cholinesterase; HbA1c, hemoglobin A1c; FPG, fasting plasma glucose; FINS, fasting insulin; HOMA-IR, homeostasis model assessment-insulin resistance; TC, total cholesterol; LDL-C, low-density lipoprotein cholesterol; HDL-C, high-density lipoprotein cholesterol; TG, triglyceride; eGFR, estimated glomerular filtration rate; UACR, urinary albumin creatinine ratio; ALT, alanine aminotransferase; AST, aspartate aminotransferase; GGT, γ-glutamine transferase; BMI, body mass index; SBP, systolic blood pressure; DBP, diastolic blood pressure.
